# The Formation of Surface Nanoparticles Enhances the Vacuum Carburizing Efficiency of 20CrMnTi Steel

**DOI:** 10.3390/nano16050305

**Published:** 2026-02-27

**Authors:** Fangpo Li, Jianjun Wang, Lihong Han, Caihong Lu, Zhuocheng Li

**Affiliations:** 1 State Key Laboratory of Oil and Gas Equipment, CNPC Tubular Goods Research Institute, Xi’an 710077, China; 2 State Key Laboratory of Digital Steel, Northeastern University, Shenyang 110819, China

**Keywords:** pre-nitriding, vacuum carburizing, microstructure, carburizing efficiency, surface characteristics

## Abstract

This work investigates the effect of pre-nitriding treatment before vacuum carburizing on the carburizing efficiency of 20CrMnTi steel. The results show that pre-nitrocarburizing significantly enhances the vacuum carburizing efficiency of 20CrMnTi steel, refines the microstructure of the carburized layer’s martensite, and promotes the precipitation of carbides. At the same carburized layer depth, the hardness and carbon content of the pre-nitrocarburized samples are significantly higher than those of the samples without pre-nitriding. Specifically, the effective hardening depth and surface hardness increase by approximately 0.15 mm and 75 HV, respectively. These improvements are attributed to the formation of loose and porous nanoscale nitride particles on the surface during the pre-nitrocarburizing process, which significantly increases the surface roughness and porosity. This surface morphology facilitates the adsorption and inward diffusion of carbon atoms during the carburizing process. This study provides some inspiration for pretreatment techniques to improve the efficiency of vacuum carburizing.

## 1. Introduction

20CrMnTi steel is widely used in the manufacturing of gears due to its excellent overall performance. To meet the demanding requirements of gears, surface modification treatments are necessary. Carburization is one of the most commonly used surface modification techniques for 20CrMnTi steel, and it significantly enhances surface hardness, wear resistance, and fatigue strength [1].

It has been found that appropriate pre-treatment before carburizing can significantly improve both the quality and efficiency of the carburizing process. With the continuous advancement of carburizing technology, the importance of pre-treatment has become increasingly recognized. The preheating serves as an effective method for regulating the uniformity of the carburized steel microstructure. Although it has a relatively minor impact on the absorption and diffusion of carbon atoms during carburizing, its role remains indispensable and has become an essential step in gear production workflows [2,3]. Pre-oxidation, nano-sizing, and pre-nitriding primarily modify the surface layer of alloy, influencing the absorption and diffusion of carbon atoms during subsequent carburization, thereby affecting the microstructural evolution of the carburized layer. The oxide protrusions formed on the workpiece surface through pre-oxidation treatment mainly aim to improve surface condition, enhance surface activity, increase carbon atom absorption, and promote diffusion. Its primary effect lies in influencing the absorption process of carburizing atoms [4,5]. However, pre-oxidation treatment has significant limitations. For instance, the pre-oxidation process varies greatly for alloy steels with different compositions. Improper selection of the oxidation process may result in an oxide film that is either too porous or too thick, severely reducing the mechanical properties of the carburized layer. Conversely, an oxide film that is too thin may significantly diminish the effectiveness of the pre-treatment [5,6]. The mechanism of surface nanocrystallization-enhanced diffusion primarily involves the formation of a nanocrystalline layer at the surface. The abundance of grain boundaries, dislocations, and twins within this layer provides diffusion pathways for carbon atoms, reducing diffusion resistance [7,8]. This process mainly influences the diffusion of carbon atoms. Surface nanocrystallization treatment is highly susceptible to limitations imposed by workpiece geometry, dimensions, and equipment constraints. For intricate, precision components such as small gears, achieving uniform nanocrystallization across all tooth surfaces or even spline areas is challenging due to complex shapes [8]. This can result in a more non-uniform diffusion layer, ultimately compromising carburizing quality.

The nitrogen introduced by pre-nitriding promotes the precipitation of nitrides or carbonitrides. These precipitates pin the austenite grain boundaries during austenitization, inhibiting grain growth and enabling the diffusion layer to maintain a fine-grained structure at higher temperatures [9,10]. Furthermore, the precipitates enhance the strengthening effect of secondary phases. In terms of surface grain refinement, pre-nitriding functions similarly to surface nanostructure by reducing the grain size in the material’s surface layer. Additionally, after nitriding treatment, steel surfaces develop abundant nitride particles, paralleling the surface modification effects achieved through pre-oxidation [11]. Compared to the larger oxide particles (approximately 20 μm) formed during pre-oxidation, nitride particles are significantly smaller (typically 10–1000 nm), giving pre-nitriding a distinct advantage in enhancing surface activity. Given these effects, pre-nitriding demonstrates significant potential as a combined pre-oxidation and surface nanostructuring technique, offering the dual benefits of enhancing surface activity and refining surface grain size. This makes it a promising and more effective pre-treatment method for carburizing. By simultaneously increasing surface activity and refining surface grains, it enhances both carbon atom absorption at the surface and promotes carbon diffusion during carburizing.

With the increasing demand for the performance of carburized parts, vacuum carburizing has gradually replaced traditional atmosphere carburizing. It has the following advantages: the carburizing temperature is high, which can improve the carburizing efficiency and save the carburizing time; the carbon potential during carburizing can be flexibly adjusted, and the carbon concentration on the surface and the thickness of the carburized layer can be easily controlled; it is not affected by oxidation, eliminating problems such as intergranular oxidation and alloy element depletion; the surface of the carburized workpiece is smooth after carburizing, saving the process of surface cleaning; the exhaust gas emission is small, the energy utilization rate is high, and it is environmentally friendly. However, the disadvantage of vacuum carburizing lies in its reliance on advanced vacuum carburizing equipment, and the manufacturing and maintenance costs of the equipment are high. Therefore, some measures need to be taken to improve the carburizing efficiency to reduce costs. Applying the grain refinement effect of pre-nitriding to vacuum carburizing allows for elevated carburizing temperatures. This endows vacuum carburizing with the advantages of high-temperature carburizing, significantly boosting carburizing efficiency and reducing production costs.

The quality and hardness distribution of the carburized layer determine its fatigue resistance and wear properties, which in turn affect the service life of the carburized workpiece. Therefore, improving the quality and hardness of the carburized layer is particularly important for enhancing the performance of carburized components. However, there has been no report on the study of how the pre-nitriding treatment alters the surface properties of steel and thereby affects the absorption and diffusion of C atoms during the carburizing process. The underlying mechanism of this effect remains unclear. In view of this, this study adopted pre-nitriding treatment to generate nano-sized nitride particles on the surface of the 20CrMnTi sample and refine the grain structure. The aim is to increase the surface adsorption area and promote element diffusion during the carburizing process, thereby enhancing the surface hardness of the carburized layer and the effective hardened layer thickness, and explore the underlying mechanisms.

## 2. Experiment

The primary chemical composition of the 20CrMnTi gear steel used in this study is presented in Table 1. The raw material is an annealed bar of 40 mm in diameter with an initial hardness of approximately 230 HV. The bars are machined into rectangular specimens measuring 10 mm × 10 mm × 25 mm and cylindrical bars with a diameter of 38 mm and a length of 200 mm, according to the experimental requirements. The surfaces are then polished smooth.

**Figure 1:**
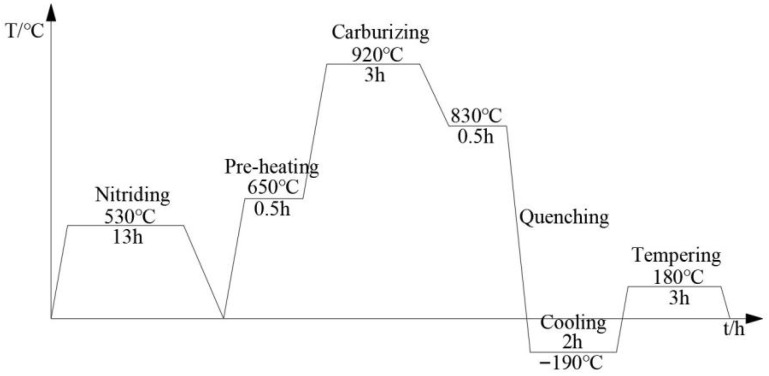
Schematic diagram of experimental process.

The samples are divided into two groups (to ensure repeatability and reliability, each group of samples consists of 5 items.): one underwent a conventional vacuum carburizing process, and the other received a pre-nitriding treatment followed by vacuum carburizing. A DB-433 vacuum furnace is used for both the nitriding and carburizing processes. The samples are loaded into the vacuum furnace and heated to 530 °C for nitriding (PN), followed by furnace cooling. Subsequently, the temperature is raised to 920 °C to initiate carburizing. After carburizing is completed, the samples are oil-quenched from 830 °C to room temperature. This is followed by cryogenic cooling in liquid nitrogen and then a low-temperature tempering at 180 °C. The vacuum nitriding medium is ammonia gas, and the ammonia decomposition rate is controlled to reach 25% by regulating the pressure inside the furnace. The vacuum carbonizing medium is acetylene gas, with a flow rate of 10 L/min. The process flow is shown in Figure 1.

Before observing the microstructure, the cross-section of the sample is ground and polished. Then, it is subjected to metallographic corrosion with a corrosive agent (4% nitric acid alcohol). Microstructure observation and analysis are conducted using an optical microscope (OM, BX53M, Olympus Corporation, Tokyo, Japan) and a scanning electron microscope (SEM, ZEISS ULTRA55, Carl Zeiss, Oberkochen, Germany). The elemental distribution of each layer in the pre-nitriding sample is analyzed by electron probe (EPMA, JXA-8530F, JEOL Ltd., Tokyo, Japan). The carburized and pre-nitriding rod-shaped samples are turned every 0.1 mm, and the nitrogen and carbon element contents of each layer of the turned chips are tested using an oxygen-nitrogen-hydrogen analyzer (ONH836, LECO Corporation, St. Joseph, MI, USA) and a carbon-sulfur analyzer (CS230, LECO Corporation, St. Joseph, MI, USA). The Vickers hardness of each group of sample layers is tested using a microhardness tester (FM700, FUTURE-TECH Corp., Kawasaki, Kanagawa, Japan), and the effective hardness value of each sample is the average of 3 measurements at different positions of the same layer depth. X-ray diffraction (X’Pert pro MRD, Malvern Panalytical, Almelo, Netherlands) is used to characterize the phase composition of the carburized samples of the two processing methods at different layer depths. The target material is Cu-Kα, the scanning angle was 30–90°, and the scanning speed is 4°/min. The XRD samples are electrolytically polished with 7% perchloric acid alcohol solution after mechanical polishing to meet the experimental requirements for the observation surface.

## 3. Results and Discussion

### 3.1. Microstructure of Nitriding Layer Analysis

**Figure 2:**
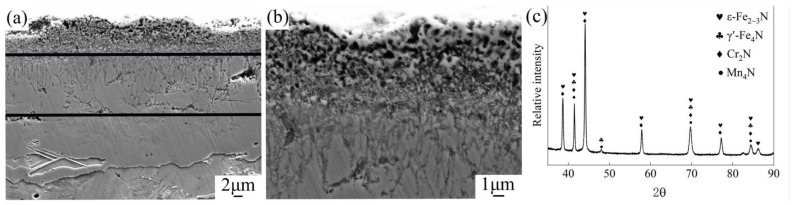
Microstructure of nitriding layer (**a**,**b**) 1000 and 5000 times of cross section by SEM; (**c**) XRD analysis of nitrided layer.

Figure 2 presents SEM micrographs and XRD patterns of the nitriding layer. The surface layer of the PN pre-treated sample exhibits a nitride compound layer approximately 25 μm thick, primarily composed of ε-Fe_2–3_N and γ′-Fe_4_N iron nitrides, along with a small amount of Mn_4_N and Cr_2_N alloy nitrides [12,13]. The nitride layer can be divided into three distinct sub-layers: the outermost layer (~5 μm from the surface) consists mainly of nanoscale nitride particles forming a loose, porous, sponge-like structure (porosity decreases with depth); the intermediate layer (5–15 μm depth) is composed of columnar crystals oriented perpendicular to the surface, with numerous grain boundaries also perpendicular to the surface. This intermediate layer is brittle and hard, and some structural damage occurred during sample preparation. The innermost layer is denser and free of obvious pores. During the nitriding process, nitrogen atoms first dissolve into the α-Fe matrix. When the nitrogen concentration exceeds the equilibrium solid solubility limit of the α/γ′ phases, a dense γ′-Fe_4_N layer forms on the sample surface. Because the diffusion rate of nitrogen within this nitride layer is very low, the nitrogen content in the γ′-Fe_4_N phase increases rapidly. Once the nitrogen concentration in γ′-Fe_4_N reaches the equilibrium level between the γ′ and ε phases, a harder and more brittle high-nitrogen ε-Fe_2–3_N phase forms at the outer surface of the nitride layer. Consequently, the outermost compound layer consists of this ε-Fe_2–3_N phase, which corresponds to the material loss observed during sample preparation. In contrast, the inner portion of the compound layer is a strong, tough, low-nitrogen γ′-Fe_4_N phase [14].

**Figure 3:**
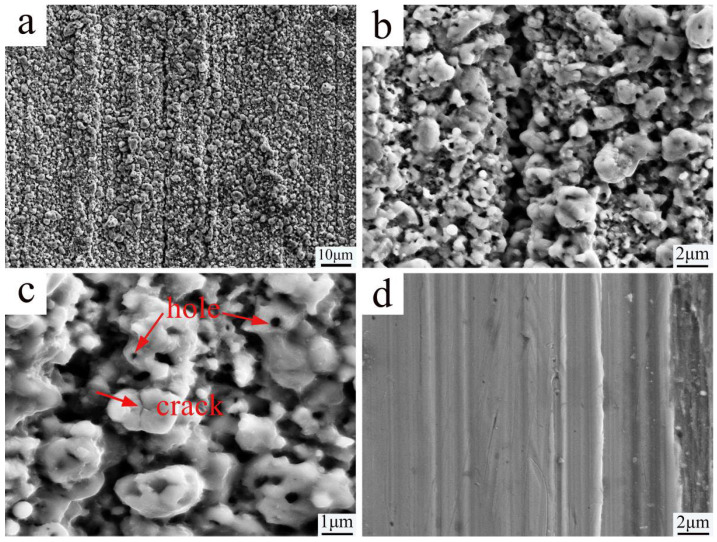
Microstructure of surface by SEM (**a**–**c**) 1000, 5000 and 10,000 times of sample with PN pre-treatment; (**d**) 5000 times of surface of sample without PN.

Figure 3 illustrates the change in surface morphology of the sample due to the PN pre-treatment. Compared to the untreated surface, the PN treatment sample surface is completely covered by nanoscale nitride particles. The original polishing marks become nearly indistinguishable, and numerous pores along with a few microcracks are present on the larger nitride particles. These features are attributed to the volume expansion caused by the aggregation and growth of nitrides during the nitriding process [15]. Studies have shown that such surface characteristics provide improved conditions for subsequent carburizing, facilitating the adsorption and diffusion of carbon atoms [4,5,16].

**Figure 4:**
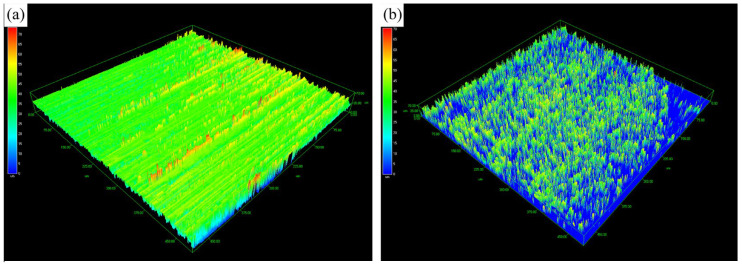
3D surface profilometry of samples. (**a**) Without PN, (**b**) With PN.

Figure 4 and Table 2 show the three-dimensional surface topography and the calculated surface area of the specimen, obtained using laser confocal microscopy. A comparison of the surface topography and area before and after pre-nitriding reveals that prior to pre-nitriding, the specimen’s surface had ridge-like protrusions mostly below 20 μm in height and about 0.5 μm in width. After pre-nitriding, the surface displays numerous uniformly distributed tree-like protrusions, with heights of around 30 μm. Surface area measurements before and after pre-nitriding are summarized in Table 2. The results indicate that the surface area increased by about 70% after pre-nitriding, with a surface area ratio of 1.701 (compared to 1.044 before pre-nitriding). The dense, uniformly distributed tree-like protrusions on the pre-nitriding specimen’s surface significantly increase its surface area. This larger surface area enhances the contact between the steel and the carburizing atmosphere during treatment, thereby increasing the adsorption of active carbon atoms per unit area and consequently facilitating the absorption and diffusion of carbon atoms.

Changes in surface roughness due to PN pre-treatment were further evaluated by contact angle measurements. Table 3 shows the measured contact angles. Here, *θ*_w_ and *θ*_p_ represent the intrinsic contact angles of the surface for deionized water and propanetriol, respectively. According to Young [17] and Wenzel [18] theories, the following relationship holds:

(1) $$\cos\theta_{w}=\gamma \times \frac{\left(\gamma^{sa}-\gamma^{sl}\right)}{\gamma^{la}}=\gamma \;\cos\theta_{Y}$$

where *θ_w_* is the equilibrium (apparent) contact angle in the Wenzel equation, *θ_Y_* is the intrinsic contact angle in the Young equation, and γ is the roughness factor of the solid surface. The roughness factor γ is defined as the ratio of the actual solid–liquid contact area to the apparent (geometric) solid–liquid contact area of a rough surface and is always greater than 1 for a rough solid surface. The terms *γ^sa^*, *γ^sl^*, and *γ^la^* represent the solid–gas, solid–liquid, and liquid–gas interfacial tensions, respectively. From the above equation, it can be seen that when the intrinsic contact angle *θ_Y_* is less than 90°, the apparent contact angle decreases as *θ_Y_* increases; conversely, when *θ_Y_* is greater than 90°, the apparent contact angle increases as *θ_Y_* increases [19].

The contact angles of both samples are greater than 90°, indicating that the surfaces are non-wetting (hydrophobic). The intrinsic contact angles (*θ_Y_*) of the PN sample for both liquids are significantly larger than those of the sample without PN, suggesting a substantial increase in surface roughness due to the PN. This is because wetting is actually a composite process involving both liquid–solid and gas–solid interfaces. When the surface is populated by a large number of nanoscale nitride particles, the liquid cannot fully penetrate the asperities, leading to air entrapment and the formation of an air film on the surface. This air film impedes the wetting of the solid by the liquid. Therefore, a larger contact angle corresponds to poorer wetting, higher surface roughness, greater pore volume, and a larger gas–solid interfacial area [20,21]. The much higher contact angle observed for the PN sample indicates that its surface roughness is significantly enhanced, which greatly promotes the adsorption of carbon atoms during carburizing. This leads to a rapid increase in surface carbon concentration, accelerates inward diffusion, and consequently improves carburizing efficiency.

### 3.2. Microstructure of Carburized Layer Analysis

**Figure 5:**
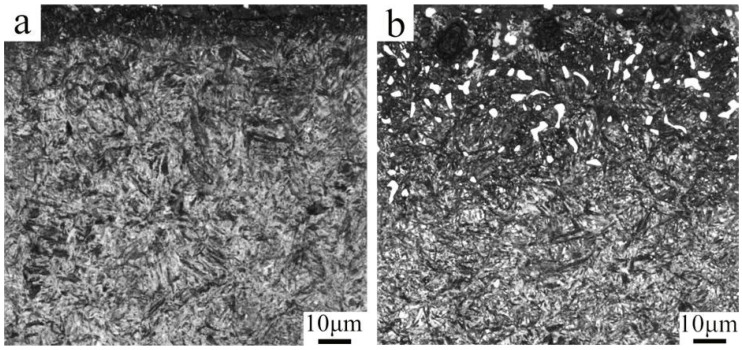
Microstructure of carburized layer by optical microscope sample. (**a**) without PN, (**b**)with PN.

Figure 5 shows the microstructure of the carburized layer in the samples without and with PN. The sample without PN exhibits almost no carbide precipitation, and the martensite in the carburized layer is relatively coarse. In contrast, the PN sample shows finer martensite and a significant amount of carbide precipitation. The carbides precipitated in the surface layer are primarily located along prior austenite grain boundaries, appearing as coarse granular and angular blocks at a depth of about 50 μm from the surface. In the sub-surface layer, the precipitates are very fine (approximately 100–600 nm) and are found on grain boundaries and within the martensitic matrix. Both the volume fraction and size of these precipitates gradually decrease with increasing depth. These observations indicate that pre-nitriding not only refines the martensitic microstructure of the carburized layer but also promotes carbide precipitation.

**Figure 6:**
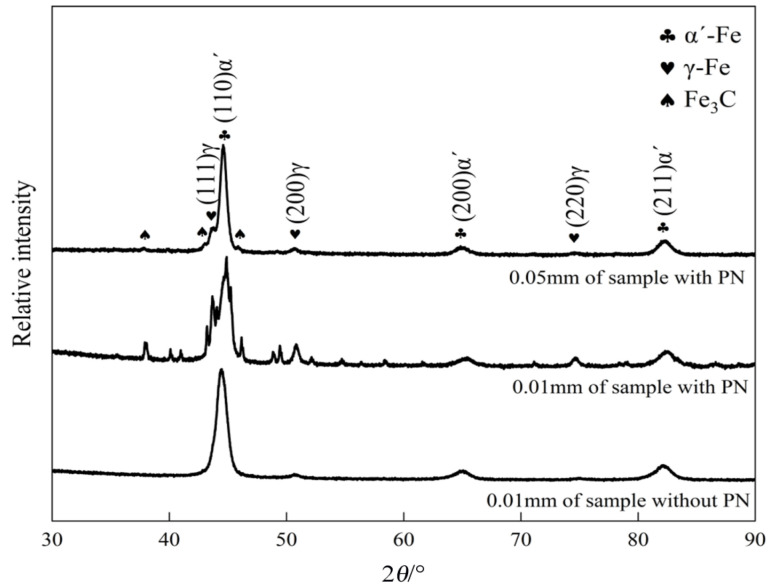
XRD analysis of the carburized layer of the samples without and with PN.

XRD results for the two groups are presented in Figure 6. In the sample without PN, the diffraction peaks of γ-Fe are very weak and no Fe_3_C peaks are observed, indicating that the matrix consists primarily of martensite (α′-Fe) with a small amount of retained austenite (γ-Fe). In the sample with PN, however, diffraction peaks of Fe_3_C and γ-Fe appear at both the surface and sub-surface, with much stronger peaks in the surface layer. This indicates that the matrix consists of α′-Fe and retained γ-Fe, and the content of γ-Fe decreases with depth. This is attributed to the high carbon content at the surface, which lowers the martensite finish temperature; consequently, after quenching, more austenite remains untransformed (retained austenite).

**Figure 7:**
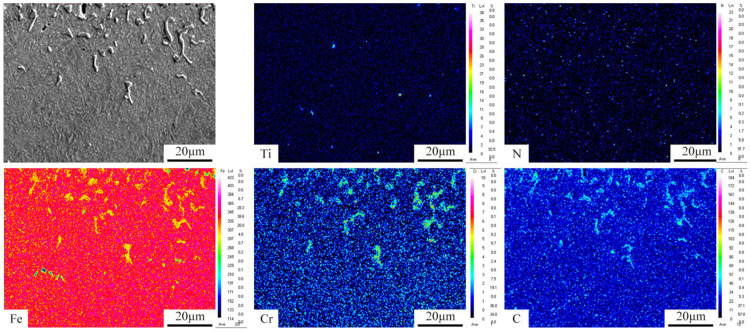
Area scanning results of EPMA for the case layer near surface of sample with PN.

Figure 7 shows an EPMA elemental map near the surface of the PN pre-treated sample. As shown in the figure, the coarse, granular, and angular cementite precipitates along the surface grain boundaries are enriched in chromium (Cr). This is because carbide-forming elements such as Cr tend to segregate at grain boundaries, and the high carbon content at the surface promotes the nucleation and growth of carbides at these boundaries. Subsequently, Cr atoms diffuse into the cementite, replacing some of the iron (Fe) atoms to form alloyed cementite. In contrast, the fine granular cementite in the subsurface layer contains no Cr. Furthermore, a few pores are observed in the carburized layer at a depth of approximately 10 μm from the surface. These pores result from nitrogen atoms combining to form N_2_ gas during the carburizing process; the gas escapes and leaves behind voids in the layer [4,13].

### 3.3. Hardness and Carbon Concentration Analysis

**Figure 8:**
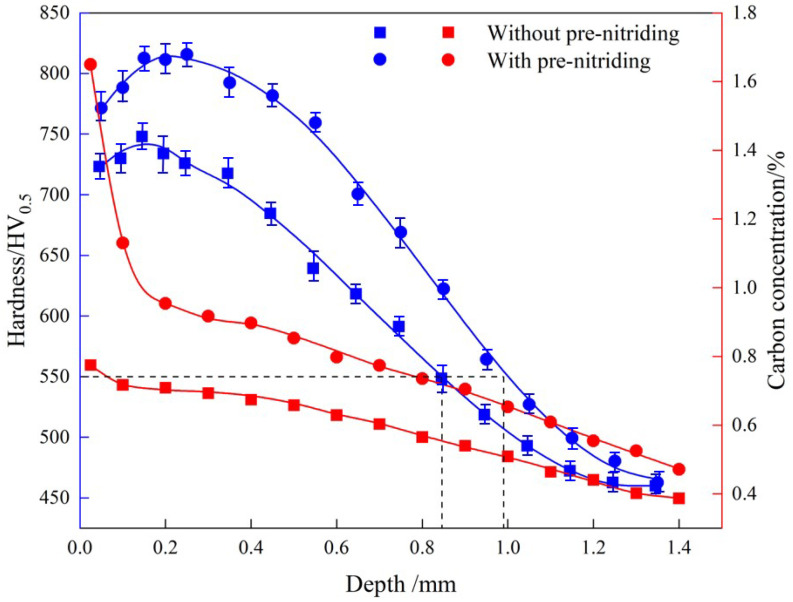
Hardness and carbon concentration of the carburized layer of sample without and with PN pre-treatment.

Figure 8 shows the hardness and carbon concentration profiles in the carburized layer for both sample groups. At the same depth, the hardness and carbon concentration of the PN sample are significantly higher than those of the sample without PN. The effective hardened layer depth and the hardness at equivalent depths in the carburized layer increased by approximately 0.15 mm (~18%, according to ISO 2639:202) [22] and 75 HV (~10%), respectively, with PN pre-treatment. Additionally, the hardness gradient in the layer became more uniform. In particular, the hardness variation within the top 0.55 mm was much smaller in the PN sample compared to the untreated sample. In the PN sample, the hardness increased from 771 HV at the surface to 816 HV at 0.1 mm, and then gradually decreased to 760 HV at 0.55 mm; in the sample without PN, the hardness increased from 722 HV to 748 HV at 0.1 mm, then dropped more rapidly to 640 HV by 0.55 mm. For carbon concentration, the sample without PN showed a steady decrease from 0.77% at the surface to 0.39% at 1.4 mm. In contrast, the PN sample exhibited a sharp drop from 1.65% at the surface to 0.95% at 0.2 mm, followed by a slower decline (similar to the untreated sample) from 0.2 mm to 1.4 mm. The higher carbon content at the surface increases the hardness of the carburized layer, which not only improves wear resistance but also suppresses the formation of surface cracks, thereby enhancing fatigue strength [23,24]. Furthermore, the greater effective case depth achieved with PN contributes to improved fatigue strength of the carburized steel [25]. By increasing the carbon content in the carburized layer, PN refines the martensitic structure and promotes carbide precipitation, thereby amplifying the fine-grain strengthening and second-phase strengthening effects [26]. As a result, both the maximum hardness and the effective case depth of the carburized layer are improved.

When carbon atoms diffuse in austenite, they preferentially migrate along crystal defects such as grain boundaries and sub-grain boundaries, because the activation energy for diffusion at these sites is about half that within the grains and the diffusion coefficient is correspondingly higher. This significantly accelerates carbon diffusion [27]. Once the carbon concentration reaches a critical level, carbides begin to precipitate at the grain boundaries and continue to grow. Since the carbon concentration is highest at the surface, carbide growth is fastest at the surface [28,29]. This explains why the carbon concentration at the surface is much higher than in the sub-surface layer. This disparity can be reduced by increasing the diffusion time or the number of boost diffusion cycles in the vacuum carburizing process.

### 3.4. Diffusion of Nitrogen Atoms During Carburizing

**Figure 9:**
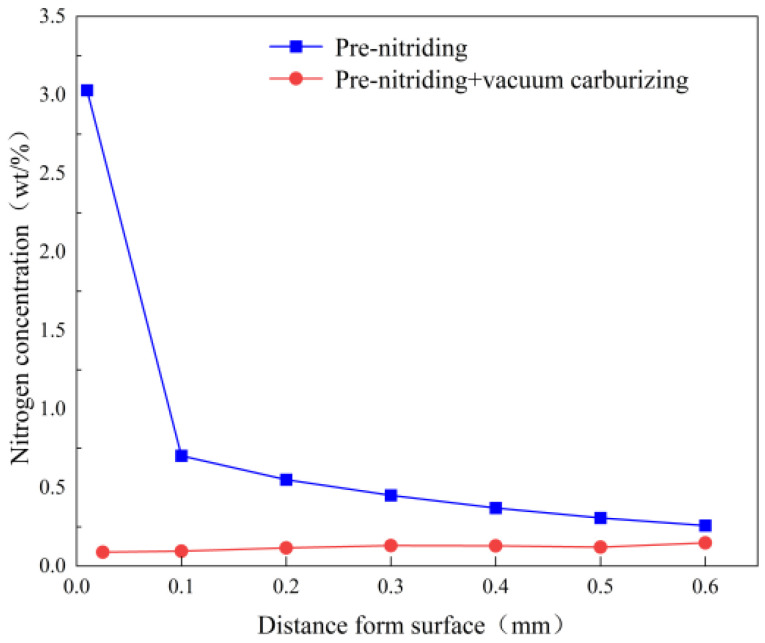
Nitrogen concentration of samples.

Figure 9 shows the nitrogen concentration profile in the PN sample, before and after vacuum carburizing. Before carburizing, the nitride layer on the surface hinders nitrogen diffusion inward, resulting in a very high nitrogen concentration in the surface layer and a much lower concentration in the deeper diffusion layer. After carburizing, however, the nitrogen content near the surface is greatly reduced (for example, at ~0.1 mm depth) and it increases with depth in the carburized layer. This phenomenon occurs because the ε-Fe_2–3_N and γ′-Fe_4_N phases formed by pre-nitriding transform and decompose into nitrogen-rich austenite during the carburizing heat. Moreover, carbon and nitrogen atoms both occupy octahedral interstitial sites in austenite and mutually repel each other; thus, the inward diffusion of carbon during carburizing pushes nitrogen atoms deeper into the material. Consequently, after carburizing, the nitrogen content increases with depth in the carburized layer. In addition, some nitrogen atoms form N_2_ gas during carburizing, creating small pores in the surface layer of the case. This outgassing also contributes to the marked reduction of nitrogen in the near-surface region after carburizing. Given that the steel contains only 0.056% Ti, only a small fraction of nitrogen atoms combined with Ti to form refractory TiN particles; thus, most nitrogen atoms remain in solid solution. In austenite, interstitial solute atoms cause lattice distortion and increase the lattice parameter. Therefore, nitrogen in solid solution expands the lattice and enlarges the interstitial sites [30]. Carbon atoms diffuse in austenite via an octahedral–tetrahedral–octahedral pathway (octahedral gap → tetrahedral gap → octahedral gap → tetrahedral gap). The enlarged interstitial sites due to dissolved nitrogen reduce the diffusion resistance for carbon atoms [31]. In other words, a small amount of nitrogen in solid solution facilitates carbon diffusion.

The above experimental results demonstrate that PN significantly improves both the carburizing efficiency and the quality of the carburized layer. There is a notable increase in the carbon content of the carburized layer, primarily due to enhanced carbon diffusion. Additionally, nitrogen atoms in solid solution induce lattice distortion, which provides some strengthening. The underlying mechanisms are as follows: first, the nitride compound layer formed on the sample surface introduces numerous nanoscale nitride particles, along with nanoscale cracks and pores, which increase surface roughness. This, in turn, greatly enlarges the surface area available for carbon atom adsorption, thereby enhancing the adsorption and inward diffusion of carbon. Second, in the subsurface layer, the presence of many grain boundaries oriented perpendicular to the surface, together with micro-pores formed by nitrogen escaping as N_2_, provides additional fast-diffusion paths. Finally, the nitrogen atoms introduced by PN into the austenite solid solution increase the lattice constant and the size of interstitial sites. The enlarged interstitial sites reduce the resistance to carbon diffusion, thereby promoting carbon diffusion.

## 4. Conclusions

The primary aim of this study was to examine the effect of pre-nitriding on the vacuum carburizing behavior of 20CrMnTi steel. Based on the results, it can be concluded that PN pre-treatment is a viable approach to improve the efficiency of vacuum carburizing for 20CrMnTi steel. The following conclusions can be drawn:

(1) Pre-nitriding treatment results in the formation of a nanoscale honeycomb-like nitride layer on the surface, significantly increasing both surface roughness and actual surface area.
(2) Pre-nitriding refines the martensitic microstructure of the carburized layer, promotes the precipitation of carbides, and leads to a high fraction of carbides within the carburized layer.
(3) Pre-nitriding significantly increases the carbon content in the carburized layer, extends the effective hardened depth of the case, raises the hardness at equivalent depths, and improves the hardness distribution in the carburized layer.

## Figures and Tables

**Table 1:** Composition of experimental steels (mass fraction, %).

C	Si	Mn	Cr	Ti	S	P	N	Fe
0.21	0.227	1.04	1.18	0.056	0.022	0.020	0.004	Bal.

**Table 2:** Face contact angle before and after pre-nitriding treatment.

Samples	Area/μm^2^	Surface Area/μm^2^	Surface Area Ratio
Without PN	202,539	211,451	1.044
With PN	202,495	344,443	1.701

**Table 3:** Contact angles of samples with and without PN pre-treatment.

Surface	Contact Angles (°)	
	*θ_w_*	*θ_p_*
Without PN	107	99
With PN	142	147

## Data Availability

The original contributions presented in this study are included in the article. Further inquiries can be directed to the corresponding authors.
